# Experimental Study of the Resistance to Influence of Aggressive Liquids on Lightweight Concrete

**DOI:** 10.3390/ma14154185

**Published:** 2021-07-27

**Authors:** Marzena Kurpińska, Elżbieta Haustein

**Affiliations:** Faculty of Civil and Environmental Engineering, Gdansk University of Technology, Narutowicza St. 11/12, 80-233 Gdansk, Poland; elzbieta.haustein@pg.edu.pl

**Keywords:** lightweight concrete, aggressive liquids, fly ash, compressive strength, absorption, granulated ash aggregate

## Abstract

In light of the scientific research, the corrosion of concrete structures is one of the main problems that may reduce their durability due to the negative impact of the natural environment. The paper analyzes the influence of the type of component on the selected properties of lightweight concrete subjected to the influence of aggressive liquids. Four concrete mixes were prepared with a granular aggregate made of foamed glass (GEGA) and aggregate made of sintered fly ash (GAA) with the use of a mineral additive: silica fly ash. The prepared lightweight concrete after one year was exposed for 60 days to the following environments: strong acid—HCl, 1% and 2% concentration, weak acid—CH_3_COOH, 1% and 2% concentration, and an aqueous salt solution of Na_2_SO_4_, 1% and 2% concentration. Then, the compressive strength was tested, and the microstructure analysis of the ready-made lightweight concrete (LWC) was performed. The degree of penetration of aggressive solutions into the cracks of the samples was assessed by means of applying 1% phenolphthalein solution. Changes in the weight of lightweight concrete samples after the test period were estimated. The obtained test results indicate that the decrease in the durability of lightweight concrete can be classified as a long-term process. Concrete with GEGA and GAA showed high resistance to aggressive environments. Moreover, the environment containing chlorides turned out to be the most aggressive, while the environment containing sulfates proved to be the least aggressive. The higher the concentration of the destructive factor was, the faster the corrosion process went. This has been proven by measuring the pH using phenolphthalein and carrying out microscopic examination. Concretes containing aggregates made of foamed glass and sintered fly ash are suitable for use both in traditional construction and in facilities exposed to an aggressive environment (e.g., in the chemical industry and at gas stations).

## 1. Introduction

Concrete is the most popular construction material all over the world, and its production and consumption are constantly increasing [1]. It has many advantages, the most important of which is relatively high durability and strength. The durability of concrete structures is measured in dozens or even hundreds of years, and at the same time, the maintenance of the structure does not generate excessive costs [2]. In recent years, a growing interest of scientists has been observed in the study of lightweight concrete, which is characterized by a lower dead load and shows better insulation than ordinary concrete [3,4]. For environmental protection reasons, the lightweight aggregates for lightweight concrete production are often obtained from recycled raw materials [5,6,7]. Besides numerous strengths, this kind of concrete also has its disadvantages [8]: it may show susceptibility to the effects of physical, physicochemical, and chemical destructive factors [9,10]. Depending on the recycled components used for the production of the aggregates, the occurrence of the unfavorable alkaline reactivity is possible, as demonstrated by the researchers [11].

Concrete corrosion can be caused by external physical or chemical factors [12]. Sometimes, it may also be caused by unwanted chemical reactions that occur in the cement matrix itself. Wrong selection of the components for the concrete mix can also result in corrosion [13]. The interaction of cement components and additives with the aggregate is of particular importance here. All these causes of corrosion are called internal aggressiveness [13,14,15]. Chemical factors are the most common trigger for the negative effects. The analysis of the causes of corrosion has been the subject of many publications [16,17,18,19]. However, in practice, each case of an aggressive environment influencing concrete should be considered individually. There are no rules that would unambiguously predict the durability of concrete under certain conditions of use. The characteristics of the corrosive environment determine how much damage it can cause and how far the corrosion may progress. These include, among others: humidity, temperature, and the type and concentration of aggressive factors [16,17,18,19,20,21]. Concrete properties are of particular importance in this case, especially liquid and corrosion resistance of hardened cement grout [22]. The most common threats include sulfate or chloride corrosion. These types of corrosion are most common in the structures exposed to groundwater, sewage, or seawater. Sulfates in groundwater are usually of natural origin but can also come from fertilizers and industrial wastewater. As the author states in [23,24], the progress of sulfate corrosion depends on the type of cation associated with the sulfate anion and on the salt concentration. The most aggressive chemical compounds include the salts of the following types: CaSO_4_, Na_2_SO_4_, K_2_SO_4_, MgSO_4_, (NH_4_) 2SO_4_, and acid—H_2_SO_4_. The defense mechanism in the case of sulfate salt penetration into the concrete is mainly based on the buffering effect of the binder components that maintain the alkaline environment of the concrete. The migration of sulfate ions with significant concentration into the concrete structure poses a risk of the formation of corrosive chemicals, in particular: ettringite (C_3_A·3CaSO_4_·32H_2_O) and gypsum (CaSO_4_·2H_2_O), as indicated by the authors [25,26,27].

The increase in the concentration of sulfate ions (SO_4_^2−^) and the decrease in the pH of the solution in the pores of the concrete result in the lack of durability of the hydrates. In the grout, in the outer layer, exposed to the environment, a solution containing gypsum appears [28,29,30]. A little deeper, in a solution with a pH of about 10, a zone containing gypsum and ettringite is formed, and in the deepest zone, not exposed to an aggressive environment, the unchanged grout is found. The first phase of sulfate corrosion improves the resistance of the concrete and increases its strength by gradually filling the capillaries and pores with corrosion products [13]. In the next phase, the gradual expansion of large-volume compounds causes unfavorable internal pressures, creating conditions for the formation of cracks. The authors of the publication [31,32,33] suggest that both ettringite and gypsum show expansive and destructive character, while in [34], the authors claim that the gypsum share is limited and ettringite expansion dominates. The researchers in [30] stated that the formation of corrosion products (gypsum and ettringite) causes an increase in the volume of concrete respectively from about 130% to 700% in relation to its initial volume. According to the author in [35,36], it is possible to increase the resistance of concrete to aggressive sulfate waters by using hydraulic or pozzolanic additives. Their presence causes a decrease in the permeability of concrete by reducing the effective ion diffusion coefficient, inter alia, by means of the binding of calcium hydroxide in the CSH phase.

Chloride corrosion causes reactions analogous to sulfates with calcium hydroxide and a decrease in the pH of the solution in the pores of concrete, which leads to its decomposition as in the case of sulfate corrosion.

The durability and quality of lightweight aggregate concrete depends not only on the type and properties of the binder, but also on the type and grain size of the aggregates. The conducted research [37,38,39] shows that lightweight aggregates are granular materials with a porous structure. Their presence provides the lightness of the final product and its good thermal insulation properties. The loose bulk density of light aggregate in a loose state should not exceed 1200 kg/m^3^ and the bulk density in a dry state should not exceed 2000 kg/m^3^. Light artificial aggregates are obtained from mineral raw materials, often recycled; their structure is transformed as a result of thermal treatment [40,41,42]. The grain size of lightweight aggregate is an important factor to be considered when designing lightweight concrete, but apart from that, there are other important properties such as petrographic composition, shape, porosity, strength, density, and water absorption. Lightweight aggregates should not be polluted in any case as they could disrupt the setting and hardening of concrete and thus lead to a decrease in strength and durability [43].

The aim of the work was to present the problem concerning the durability of lightweight concrete (LWC) exposed to the influence of harmful factors of the external environment. The main task was to link the impact of destructive factors on lightweight concrete in general, and specifically on its durability. For this purpose, four different concrete mixes were prepared containing granular foamed glass aggregate (GEGA) and sintered fly ash aggregate (GAA). The lightweight concrete samples were exposed to aggressive aqueous chemical solutions of 1% and 2% concentrations of hydrochloric acid (HCl), acetic acid (CH_3_COOH), and sodium sulfate (VI) (Na_2_SO_4_).

## 2. Materials and Methods

### 2.1. Materials Characterization

Following [44], CEM I 42.5R Portland cement (Lafarge, Kujawy, Poland) and fly ash (Dolna Odra, Szczecin, Poland) were used to perform the tests. The chemical composition and physical properties of the binders are shown in Table 1.

**Figure 1:**
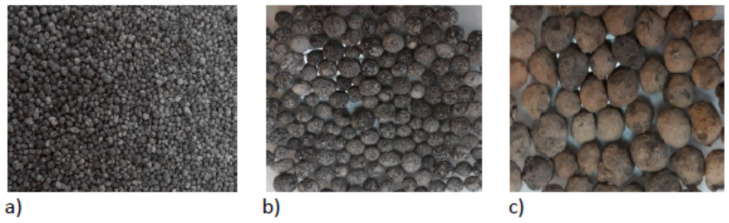
Structure of the aggregates used in the research: (**a**) granulated expanded glass aggregate GEGA 2 mm, (**b**) granulated expanded glass aggregate GEGA 4 mm, (**c**) granulated fly ash aggregate (GAA) 8 mm.

The properties of the grain size of GEGA 2 and 4 and GAA 8 mm (Figure 1) were examined, and the results are presented in Table 2. Physical properties, distribution of GEGA and GAA, and their pore structure are shown in Table 3. The GAA 8 mm grain is characterized by a significantly lower porosity and a higher density than the GEGA grain. The GEGA 2 mm outer grain surface shows much larger quantity of pores with smaller diameters than that of the GEGA4 mm. The GEGA 4 mm outer grain surface is also an open structure that is quite crisp and cracked, which can lead to LWC damage. The empty spaces and pores of the GEGA grain may be filled with liquids or slurry. Therefore, it is very important to protect the grains of light aggregate by impregnating them with cement paste. Proper impregnation allows for obtaining desirable properties and working connection of the aggregate grains.

### 2.2. Mix Proportion and Mixtures

Four concrete mixes (R1 ÷ R4) were designed. The concrete composition was designed assuming a variable water/cement ratio and the type of lightweight aggregate. The main parameter considered in the corrosion resistance analysis was the water/binder ratio, which changed along with the content of cement and ash in the recipes and constituted 0.5 (R1), 0.45 (R2), 0.4 (R3), and 0.35 (R4). For all recipes, only artificial lightweight aggregates were utilized. In recipes for R1 and R2, GEGA2 mm and GEGA4 mm were used in the amount of 25% of the total volume of lightweight aggregate and GAA 8 mm in the amount of 50% of the total volume of the aggregates. In the R3 and R4 recipes, only GEGA 2 mm and GEGA 4 mm were used in the amount of 50% of the total aggregate volume. The formulas R1 and R4 contained fly ash as an additive fly ash. A chemical admixture, superplasticizer FK 63.30 (MC Bauchemie, Środa Wielkopolska, Poland), was applied in the lightweight concrete mixtures. The amount of admixture and the amount of mixing water in the four prepared concrete mixes was maintained at 0.8% of the cement weight. The composition of the mixtures is presented in Table 4.

The proportions of the particular amounts of concrete components, given in Table 4, were dosed in the following order: cement, fly ash, chemical admixture mixed with some amount of mixing water. Light aggregates were added to the ready-made cement paste. The mixes designed and made according to recipes (R1 ÷ R4) were mixed for approx. 3 min in a planetary mixer with the capacity of 75 dm^3^ and then the remainder of the water was added. To test the compressive strength and the resistance of lightweight concrete to the influence of aggressive liquids, cubes with the dimensions of 100 mm × 100 mm × 100 mm were formed in accordance with the requirements [45]. After 24 h, the samples were disassembled and placed for a period of one year in a room with a temperature of 20 ± 2 °C and air humidity above 95%. After the specified maturation period, assuming that chemical reactions had taken place and that the concrete structure had been fully formed, the concrete corrosion tests began.

### 2.3. Tests

#### 2.3.1. The Penetration Depth of Aggressive Liquids

The penetration depth (mm) of aggressive liquids into the structure of the samples was carried out after 30 and 60 days of exposure to aggressive corrosive solutions as required [46]. The penetration depth of aggressive corrosive solutions into the concrete structure with various types of lightweight aggregates was examined based on fractures obtained after the mechanical splitting test (splitting the samples into two halves). The fracture surfaces were tested after being moisturized with distilled water and after applying a chemical indicator—1% phenolphthalein solution. The phenolphthalein test was performed in accordance with the [47] standard. An alcoholic phenolphthalein solution turns red-violet at pH values higher than pH 8.5 ÷ 9.5. The pH value of fresh concrete remains within the range of 11.8 ÷ 12.6, while if the alkalinity drops to pH < 9.5, it causes the loss of stability of the passive layer on the steel elements. The state of reduced LWC alkalinity may lead to a partial loss of the protective properties of concrete and the risk of steel corrosion.

#### 2.3.2. LWC Absorption after Exposure to Corrosive Environments

The results of changes in the average absorption of LWC samples, having been previously stored in water and used as a comparative material, subjected to three types of corrosive environments of different concentrations (1% and 2% HCl, 1% and 2% CH_3_COOH and 1% and 2% Na_2_SO_4_), were assessed after 30, 45, and 60 days from the moment of immersion in corrosive solutions. Due to the shortened test time—60 days, the concentration of 1% and 2% of aggressive liquids was selected. The choice of corrosive solutions (HCl, CH_3_COOH and Na_2_SO_4_) is conditioned by an attempt to assess the corrosion risk in the case of lightweight concrete including the mechanism of the influence of ions of strong and weak acids and salts, ions occurring in the natural environment (e.g., in water, in sewage and soil surrounding the structure or concrete element).

The weight of the corrosion-treated samples and reference samples stored in water after drying in the temperature of 105 °C was compared. Each obtained result is the arithmetic mean of the tests of three samples.

#### 2.3.3. Corrosion Resistance as a Change in the Density of LWC

Concrete density test was conducted by means of the volumetric method. Before the start of the tests, three specimens of each variant of LWC R1–R4 was dried at the temperature of 105 °C until they reached a steady mass state according to [48]. Each obtained result is an arithmetic mean of three independent mass measurements. Drying the test samples to a state of a constant mass at 105 °C had an impact on the process of the evaporation of free, physio-chemically bound water and crystallization water stored in the empty spaces of the lightweight concrete structure because of the action of the selected corrosive environments. Volume density was calculated based on the following pattern:

(1) $$\rho =\frac{m}{V},$$

where *ρ* is the sample’s volume density; [g/cm^3^]; *m* is the mass of the specimen dried at the temperature of 105 °C [g]; and *V* is the volume of the specimen [cm^3^].

#### 2.3.4. Corrosion Resistance as a Change in the Compressive Strength of LWC

The corrosion resistance of concrete, containing various types of lightweight aggregate, was determined as the ratio of the compressive strength of the samples stored in corrosive solutions (1% and 2% HCl, 1% and 2% CH_3_COOH, and 1% and 2% Na_2_SO_4_) to the compressive strength of samples stored in water of a constant temperature of 20 ± 2 °C. At the time of commencement of the corrosion resistance tests, the samples were one year old. Earlier throughout the 1-year period, all samples had been stored in a room with a constant temperature of 20 ± 2 °C and air humidity of 95%. The compressive strength test of each LWC type after 30, 45, and 60 days was done by means of an Advantest 9 Controls machine (Advantest 9, Controls, San Maurizio Canavese, Italy) with a maximum pressure force of 3000 kN, according to [49]. Each obtained result was the arithmetic mean of the tests of three samples.

#### 2.3.5. Microstructure (SEM)

The morphology of the structure of the lightweight concrete samples after 60 days of exposure to corrosive solutions was determined using a scanning electron microscope (SEM, HITACHI Tokyo, Japan, TM3030 Manual Stage version, Model 55E-0015). The research was carried out to determine the degree of the damage to the internal structure of the samples including the observation of the precipitated corrosion products. Before assessing the microstructure (SEM) of the selected concrete surfaces with the use of lightweight aggregate (GEGA and GAA), cubic samples with dimensions of 20 mm × 20 mm × 20 mm were cut out. The above-mentioned samples were ground and cleaned of dust.

## 3. Results and Discussion

### 3.1. Penetration Depths of Aggressive Liquids in LWC after Exposure to Corrosive Environments

**Figure 2:**
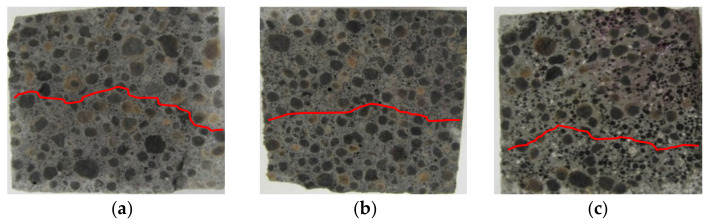
A view of the fractures of the surface of LWC (R1) subjected to the influence of: (**a**) 2% HCl, (**b**) 2% CH_3_COOH, (**c**) 2% Na_2_SO_4_.

**Figure 3:**
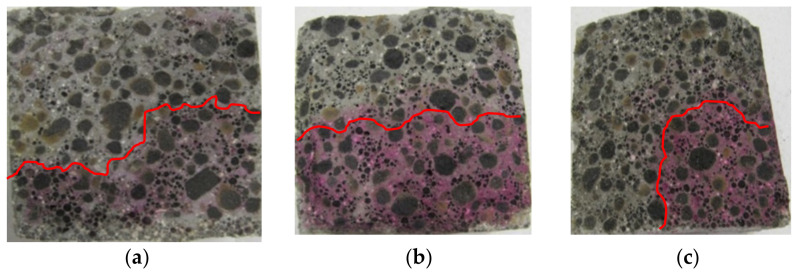
A view of the fractures of the surface of LWC (R2) subjected to the influence of: (**a**) 2% HCl, (**b**) 2% CH_3_COOH, (**c**) 2% Na_2_SO_4_.

**Figure 4:**
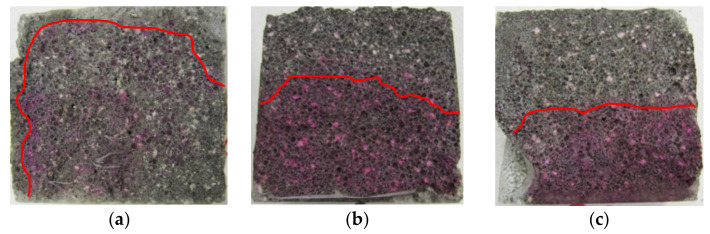
A view of the fractures of the surface of LWC (R3) subjected to the influence of: (**a**) 2% HCl, (**b**) 2% CH_3_COOH, (**c**) 2% Na_2_SO_4_.

**Figure 5:**
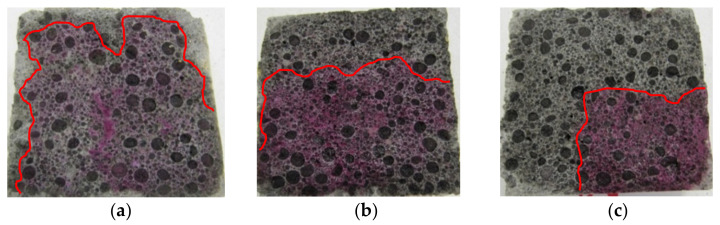
A view of the fractures of the surface of LWC (R4) subjected to the influence of: (**a**) 2% HCl, (**b**) 2% CH_3_COOH, (**c**) 2% Na_2_SO_4_.

An example of a general view of the fractures of the surface of LWC samples (R1–R4) moisturized with a 1% solution of phenolphthalein, is shown in Figure 2, Figure 3, Figure 4 and Figure 5. The visible fractures show the depth of penetration of aggressive liquids as a result of the influence of corrosive environments after 30, 45 and 60 days.

**Figure 6:**
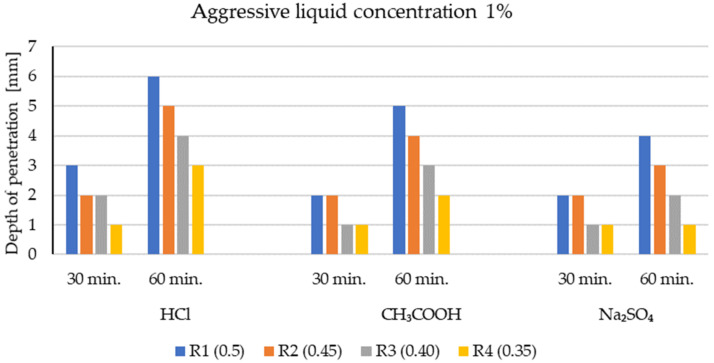
The depth of penetration of aggressive liquid concentration of 1% into the structure of LWC (R1 ÷ R4).

**Figure 7:**
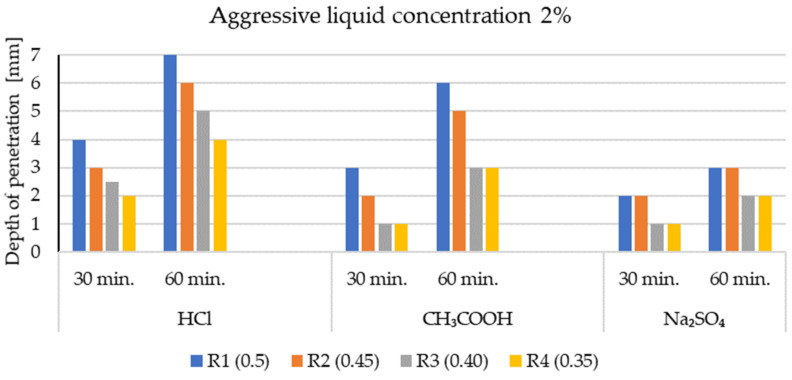
The depth of penetration of aggressive liquid concentration of 2% into the structure of LWC (R1 ÷ R4).

The penetration depth of corrosive solutions (mm), depending on their concentration in the structure of the test series (R1 ÷ R4), is presented in Figure 6 and Figure 7. The test results are the arithmetic mean obtained from the measurements of three single test samples. The tests were carried out after 30 and 60 minutes of exposure to the environment of aggressive chemical compounds.

On the basis of the examples (Figure 2, Figure 3, Figure 4 and Figure 5), it can be observed that under the influence of the corrosive environments (HCl, CH_3_COOH and Na_2_SO_4_) with the selected levels of concentration (1% and 2%) and the moisture present in the pores of the concrete, completely immersed in an liquid, the subsurface layer of concrete undergoes a gradual process of penetration by aggressive corrosive liquids. The front of the penetration of aggressive solutions gradually moves into concrete, and the main reaction taking place in this process is the reaction of corrosive solutions with calcium hydroxide dissolved in the concrete pore liquid. As a result of this reaction, Ca(OH)_2_ leaches out, which reduces the alkalinity of the concrete (neutralizes the concrete), which in turn leads to an increase in the degree of the penetration of aggressive liquids into the structure of LWC, depending on its composition.

The phenolphthalein indicator (Figure 2, Figure 3, Figure 4 and Figure 5) showed no change in color, which indicates a neutralization (pH < 8.2) of the fracture surfaces of the LWC samples tested. The analysis of the penetration of aggressive liquids showed the influence of the water/binder ratio (w/b) on the penetration depth of aggressive liquids. In the case of LWC with the composition as in R1 (w/b = 0.5) (Figure 2) and the highest w/b ratio, it was noticed that the penetration depth of aggressive liquids was the greatest, and after 60 days, it constituted from 3 mm (2% Na_2_SO_4_) to 7 mm (2% HCl). As the w/b index decreased, a decrease in the depth of liquid penetration was also visible. Therefore, for R2 (w/b = 0.45) (Figure 3) the penetration depth constituted from 3 mm to 6 mm, whereas for R3 (w/b = 0.40), it was from 2 mm to 5 mm (Figure 4). In the case of R4 (w/b = 0.35) (Figure 5), the penetration depth of aggressive liquids was the lowest, and after 60 days, it varied from 2 mm to 4 mm. In the case of LWC R2 and R3 not containing fly ash in the composition, subjected to a 2% HCl environment for 60 days, a bright pink color of the surface covered with phenolphthalein was visible, which indicated a change in the pH of the surface within the range from 8.2 to 10.5 and a decrease in the alkalinity of the cement matrix in the composition and with it, the loss of passive capabilities (Figure 3). The decrease in pH was most visible in the case of the interaction of 2% HCl (Figure 3a). The highest pH was observed in the case of R1 (with GAA) and the least in the case of R4 (without GAA). Testing the impact of the two remaining corrosive solutions: 2% CH_3_COOH and 2% Na_2_SO_4_, after 60 days of exposure of the samples, there was no risk of loss of LWC alkalinity pH > 10.5. The open porosity of the lightweight aggregate and the presence of pozzolanic mineral additives appeared to have an influence on the penetration depth of aggressive liquids. The presence of fly ash in the R4 (Figure 5) mixture after a one-year maturation period before the corrosion tests suggests the formation of the voids in the structure of lightweight concrete and filling them tightly with the products of the hydration process, which limit the penetration of the corrosive environment and the degradation of the cement matrix. This was also confirmed by other researchers in their scientific works [3,36,50].

It was stated that the penetration depth of corrosive solutions, regardless of their type and concentration, depends on the composition of the designed LWCs (Table 4). The water/cement or water/binder ratio has a particular impact on the protection against the penetration of aggressive liquids. The lower the water/cement coefficient, the shallower the liquid penetration will be. The depth of penetration of the liquid did not exceed 7 mm after 60 days of the exposure of the samples to aggressive liquid environments.

### 3.2. LWC Absorption after Exposure to Corrosive Environments

**Figure 8:**
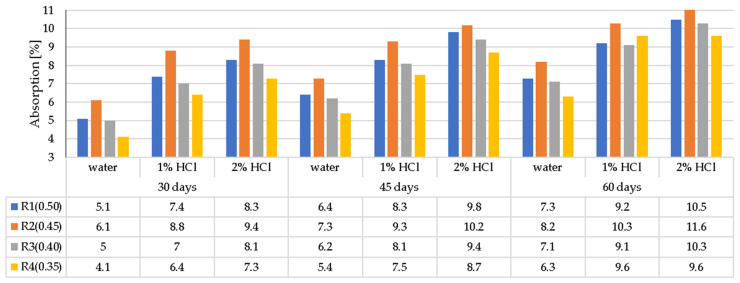

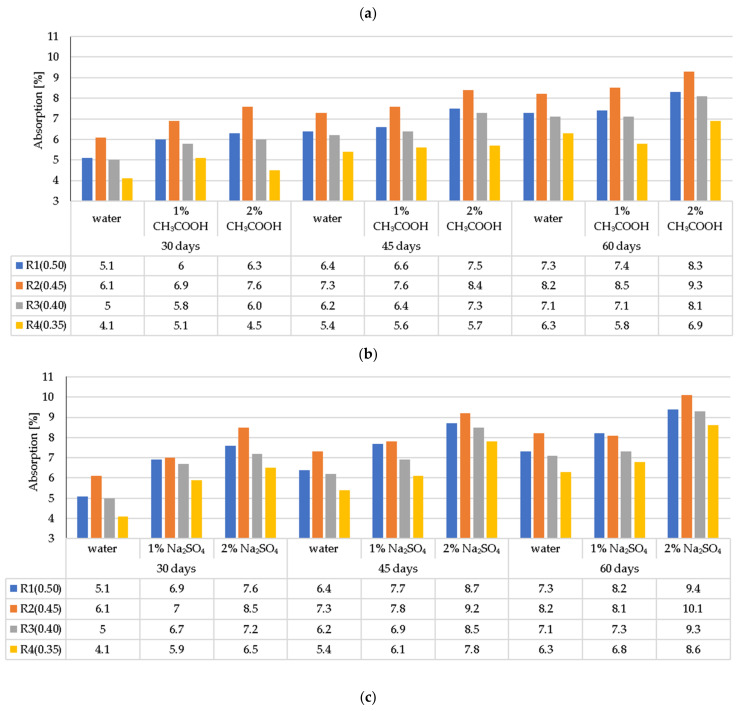
Average absorption of LWC, subjected to the following environments: (**a**) HCl, (**b**) CH_3_COOH, (**c**) Na_2_SO_4_.

The results of changes in the average absorption of LWC samples, having been previously stored in water and used as a comparative material and samples, subjected to three types of corrosive environments of different concentration (1% and 2% HCl, 1% and 2% CH_3_COOH, and 1% and 2% Na_2_SO_4_), were assessed after 30, 45, and 60 days from the moment of immersion in corrosive solutions. The weight of the corrosion-treated samples was compared with the weight of samples immersed in water. In both cases, samples had been dried in the temperature of 105 °C before weighing. The test results for LWC absorption after exposure to corrosive environments are presented in Figure 8a–c.

The obtained results of the tests and the analysis of the average values for changes in LWC absorption indicate an increase in aggressive liquid absorption in all environments compared to the water absorption of samples stored in water. In general, the LWC water absorption remained between 4.1 and 11.6%. The lower water/binder (w/b) ratio of LWC in the tested samples was assumed, and a lower water absorption was observed. In the case of w/b = 0.35, it did not exceed 9.6% in any aggressive environment after 60 days. The positive effect of the fly ash content was also visible, which limits the penetration of both water and aggressive liquids. For example, for LWC samples R1 (0.5) with a mixture of GEGA aggregate (25%, 2 mm grain size and 25%, 4 mm grain size) and GAA (50%, 8 mm) and with the addition of fly ash, liquid absorption in most cases was equal to or slightly lower than for LWC R2 (0.45) containing no fly ash and almost the same as for LWC R3 (0.4). This fact draws attention regarding its economic and environmental importance. Due to the limitation of the penetration of aggressive liquids, it is possible to use fly ash as a cement substitute in the amount of min. 20% of cement weight. Regarding the composition of LWC R2 with the mixture of GEGA and GAA aggregates in the same proportions as in the case of LWC R1, the lack of fly ash and an increase in the amount of CEM I 42.5 R by 50 kg/m^3^ did not change the water absorption of the tested samples despite an increase in the concentration of corrosive solutions during the testing period. In the case of R1 and R2, the average absorption values after 60 days of exposure in a 2% solution ranged from 10.5% to 11.6% (HCl), from 8.1% to 9.6% (CH_3_COOH) and from 9.4%, respectively, up to 10.1% (Na_2_SO_4_). In the case of LWCs R3 (0.4) and R4 (0.35) made of GEGA 2 mm and GEGA 4 mm lightweight aggregates, each constituting 50% of the aggregate volume, the LWC R4 composition additionally uses fly ash in the amount of 20% of the cement mass. It was observed that because of the additional amount of fly ash, the water absorption of the R4 samples decreased by about 10%. When analyzing the test results, it can be observed that even though the GEGA aggregates were characterized by a much higher open porosity than the 8 mm GAA aggregate, the absorbability of LWC R3, R4 compared to LWC R1, R2 samples, stored in water and aggressive environments for 60 days, did not differ by more than 1% in any case. The observed differences in changes in the water absorption of the tested concrete samples (R1–R4) with lightweight aggregate depend on the exposure time and concentration of the selected corrosive environments, and on the physical properties of GEGA and GAA aggregates (Table 3). GEGA aggregate grains (4 mm) have the highest open porosity amounting to 42% in relation to the grain porosity of 2 mm for the GEGA aggregate and 8 mm for the GAA aggregate. Their open porosity constituted 37%. The use of 4 mm aggregate grains (GEGA) in various proportions increased the penetration depth of corrosive solutions into the concrete microstructure. Considering the chemical properties (Table 2), GEGA grains showed higher CaO content of 14.9% compared to the GAA grains of 4.5%. Calcium oxide (CaO), which is a frequent component of lightweight aggregate grains and occurs in cement grains and mineral additives, dissolving in the mixing water environment, takes part in the binding process, creating hydration products (e.g., C-S-H and Ca (OH)_2_ phase). Ca (OH)_2_ shows the greatest susceptibility to aggressive corrosive environments in the cement matrix. Its dissolution enables the migration of aggressive chemical liquids into the LWC structure. The result of this phenomenon is a relatively high water absorption, which must be compensated by the increased cement content or the addition of fly ash.

### 3.3. Changes in the LWC Density after Exposure to Corrosive Environments

**Figure 9:**
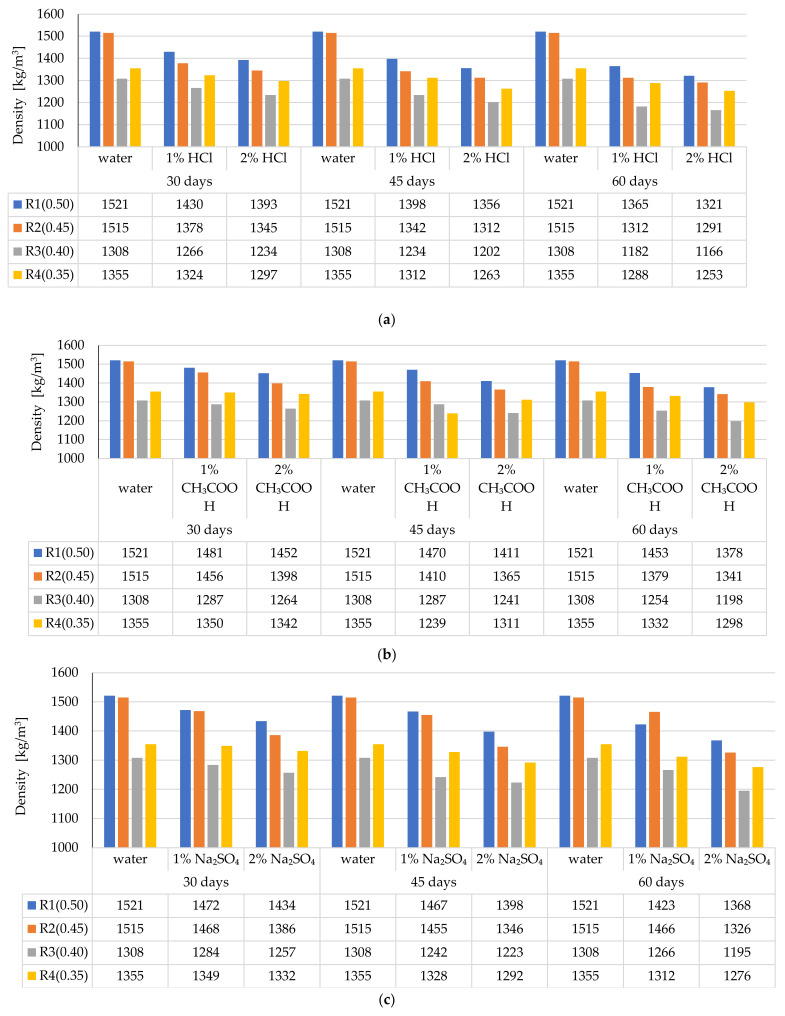
Average density of LWC depending on the type and concentration of the corrosive solution and the time of exposure: (**a**) HCl, (**b**) CH_3_COOH, (**c**) Na_2_SO_4_.

The density of LWC, depending on the exposure time to corrosive solutions compared to the density of the samples stored in the air-conditioned chamber, is presented in Figure 9a–c. The presented results of the density are the mean of the three single test results defined in the adopted testing periods.

The greatest changes in the mass and, consequently, in the density of the samples were noted when the LWC samples were exposed to a 2% HCl solution for 60 days. In this case, greater weight loss occurred in the LWC R1 and R2 group, and in the LWC R2 group, where the weight loss constituted 15% while in the case of LWC R1, it was only 13%. Lower weight loss in the case of LWC R2 resulted from the use of CEM I with the addition of fly ash. In this case, the ash was a cement substitute in the amount of 20%. Although in the case of LWC R1, the w/b ratio = 0.50 was higher than in the LWC R2 w/b = 0.45, and the weight loss in the HCL environment was 2% lower. In the case of LWC R3 and R4, whose w/b were 0.4 and 0.35, respectively, lower weight loss was recorded for the LWC R4. The results of the tests were influenced by the low w/b ratio = 0.35. In the case of LWC R4, fly ash was an addition of 20% to the cement mass. For the LWC R3, the weight loss constituted 11%, while for the LWC R4, the weight loss for the samples stored in 2% HCl reached 8%. When LWC samples were stored in the environment of 2% CH_3_COOH, the weight loss of the samples was noted in the range of 4–11%. On the other hand, the weight loss of the LWC samples treated with 2% Na_2_SO_4_ ranged from 6% to 12%.

### 3.4. Compressive Strength of the LWC after Exposure to Corrosive Environments

**Figure 10:**
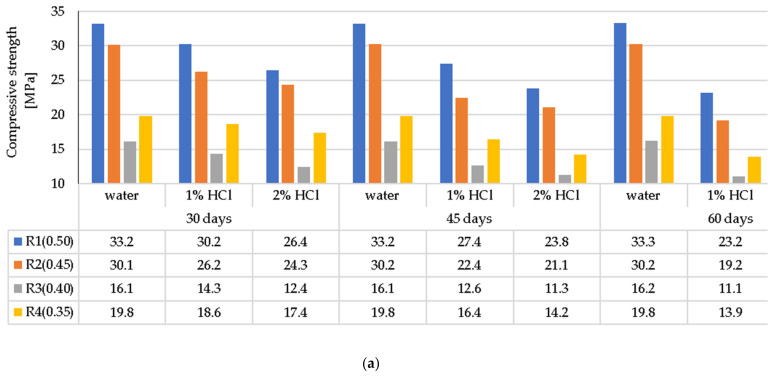

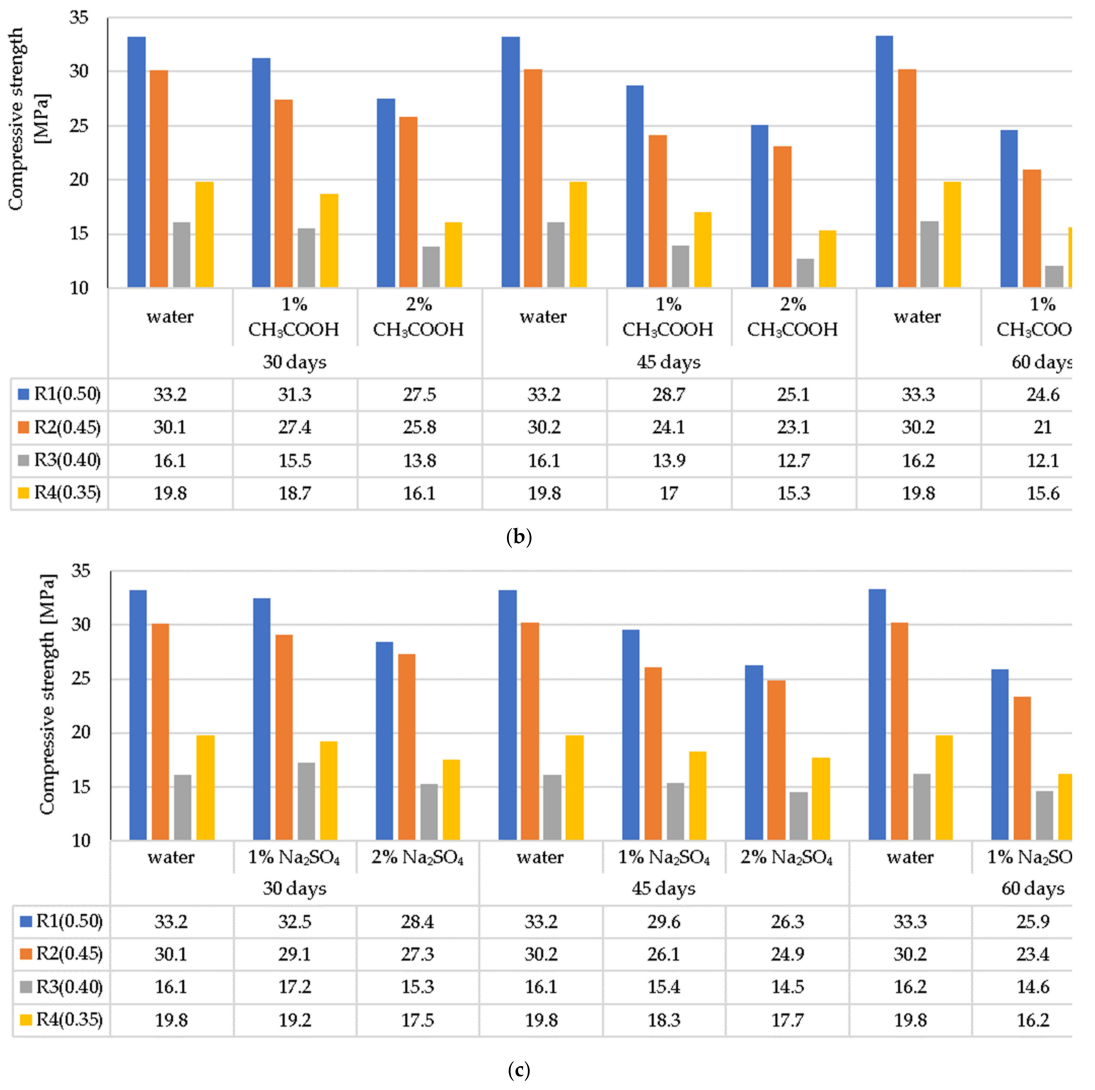
Average compressive strength of LWC depending on the type and concentration of corrosive solution and the time of exposure to: (**a**) HCl, (**b**) CH_3_COOH, (**c**) Na_2_SO_4_.

The compressive strength of LWC (R1÷R4), depending on the exposure time to corrosive solutions compared to the compressive strength of the samples stored in the air-conditioned chamber, is shown in Figure 10a–c. The presented results of the compressive strength are the mean (f_cm_, cube) of the three single test results (f_ci_, cube) defined in the adopted research terms.

The greatest decrease in LWC strength (R1 ÷ R4) was noted when the LWC samples were exposed to a 2% HCl solution for 60 days. In this case, greater decrease in weight concerned the LWC R1 and R2 groups, and, in particular, the LWC R2, where the decrease in compressive strength constituted 43%, while in the case of LWC R1, the compressive strength decreased by 41%. Lower weight loss in the case of LWC R2 resulted from the use of CEM I with the addition of fly ash. The sealing of the structure limited the influence of the aggressive liquid and the damage to the cement matrix. In the case of LWC R1, the ratio w/b = 0.50 was higher than LWC R2 w/b = 0.45, and the decrease in compressive strength in the HCl environment was 2% lower. In the case of LWC R3 and R4, whose w/b were 0.4 and 0.35, respectively, the decrease in compressive strength of the samples stored in 2% HCl for 60 days was the same in both cases and amounted to 38%. In the case of LWC R4, with an addition of 20% fly ash to the cement mass, the compressive strength increase was not observed. In the case of the LWC, the main factor influencing the strength of the LWC was the crush strength of the LWA. When the LWC samples were exposed to 2% CH_3_COOH for 60 days, the weight loss of the samples was in the range of 28–38%. On the other hand, the decrease in compressive strength of LWC samples in 2% Na_2_SO_4_ ranged from 15% to 34%. The obtained test results of the average compressive strength in a function of time and the impact of selected corrosive environments, despite the lower ratio (w/c and w/b), suggest dissolution of the cement paste covering and filling the pores of light aggregate grains, and as a result, enable crystallization (including penetration) of corrosive compounds (Figure 2, Figure 3, Figure 4 and Figure 5) in the voids of the lightweight concrete structure.

**Figure 11:**
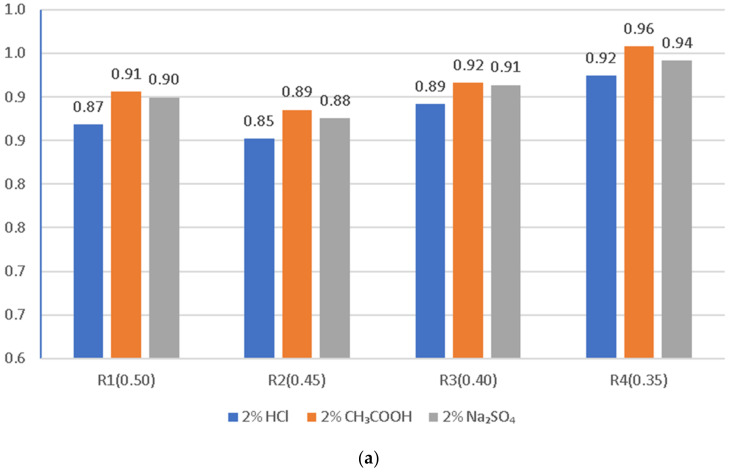

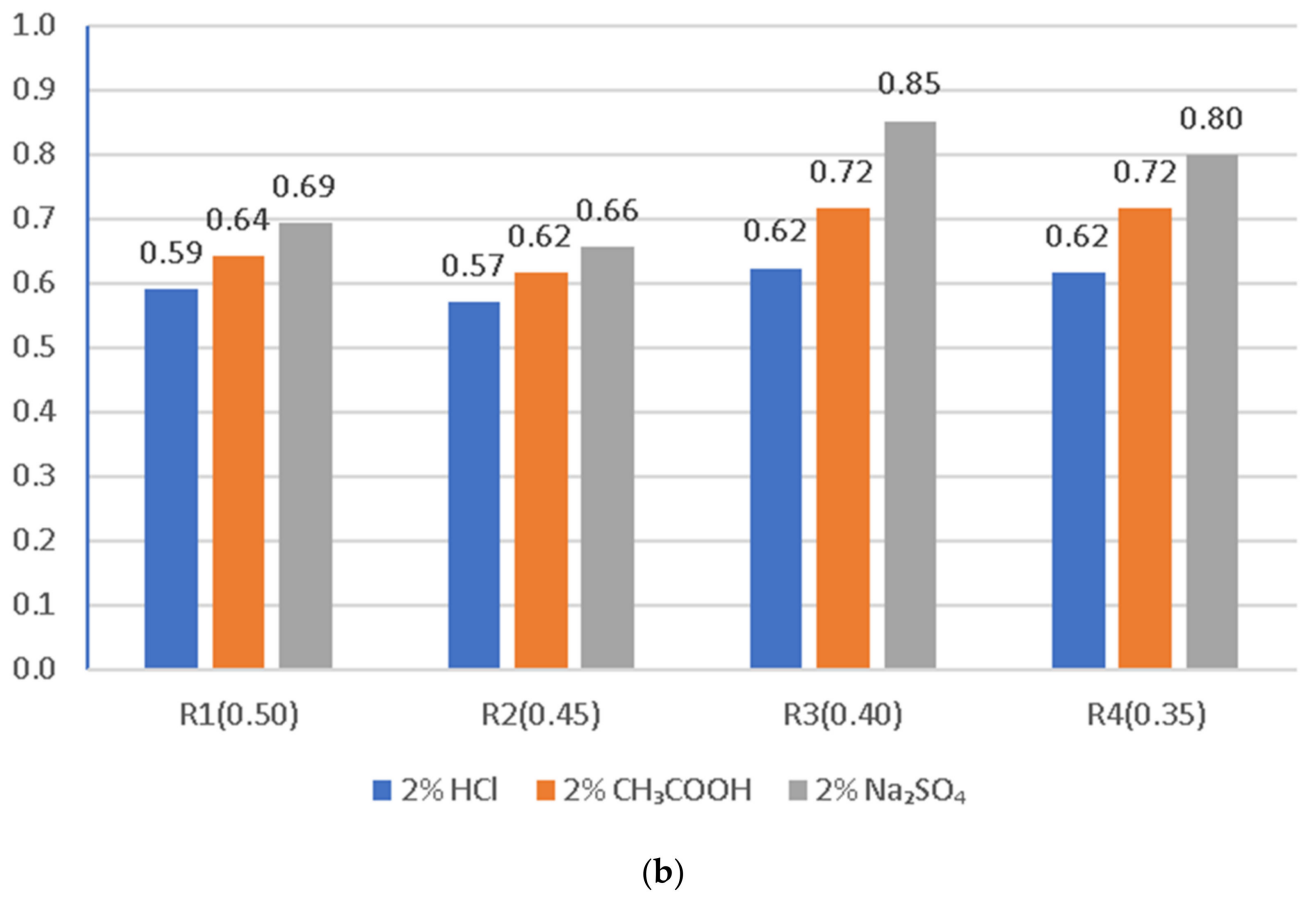
Corrosion resistance of LWC determined on the basis of changes (**a**) in the mass and density and (**b**) in the compressive strength of the samples in aggressive environments of 2% concentration after 60 days of exposure.

Graphical interpretation of the corrosion resistance coefficient of LWC (R1 ÷ R4) in the given corrosive solutions based on weight loss and compressive strength in environments of 2% concentration after exposure for 60 days is presented in Figure 11a,b.

The greatest decrease in the compressive strength of the LWC was visible after 60 days of exposure to 2% solutions of aggressive liquids described in this study. A summary of the test results is presented in Figure 11a,b as a determination of the corrosion resistance of each individual LWC type (R1–R4). The lowest resistance to the environment of 2% HCl compared to the samples stored in water was noted for LWC R1 and R2, where the decrease in compressive strength was 41% and 43%, respectively. In the case of LWC R3 and R4, the decrease in compressive strength was respectively lower and amounted to 38%. In the case of the influence of weak acid (CH_3_COOH) with a concentration of 2%, the decrease in compressive strength for LWC R1 and R2 was from 28% to 38%. Exposure of LWC to a salt solution—sodium sulfate (VI), after 60 days, caused a decrease in the strength of LWC R1 and R2, and constituted respectively 31% and 34%, while in the case of R3 and R4, the decrease in compressive strength was observed at the level of 15% and 20%, respectively. The analysis of the test results proved the significant influence of the components used on the mechanical properties of LWC.

### 3.5. The Microstructure of Concrete with LWA (GEGA and GAA) after Exposure to Corrosive Environments

**Figure 12:**
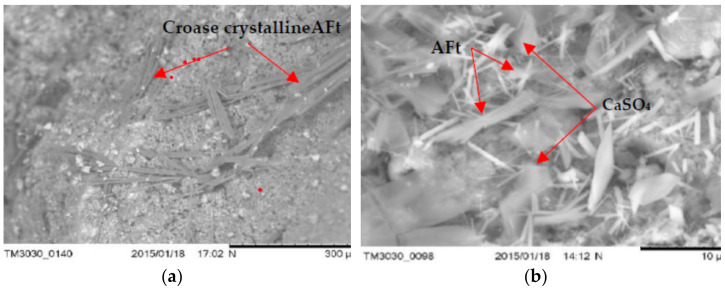
SEM. General view of the microstructure after exposure to an environment with 2% HCl (60 days) of: (**a**) LWC R1 with GEGA 2 mm 25%, GEGA 4 mm, 25%, and GAA 8 mm, 50%, and with the additive: fly ash (**b**) LWC R2 with GEGA 2 mm, 25%, GEGA 4 mm, 25%, and GAA 8 mm, 50% without fly ash.

**Figure 13:**
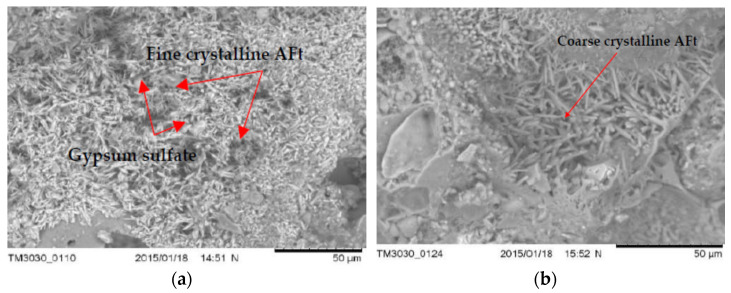
General view of the microstructure after exposure to the environment with 2% CH3COOH (60 days) of: (**a**) LWC R1 with GEGA 2 mm, 25%, GEGA 4 mm, 25%, and GAA 8 mm, 50% and with the additive: fly ash (**b**) LWC R2 with GEGA 2 mm, 25%, GEGA 4 mm, 25%, and GAA 8 mm, 50% without fly ash.

**Figure 14:**
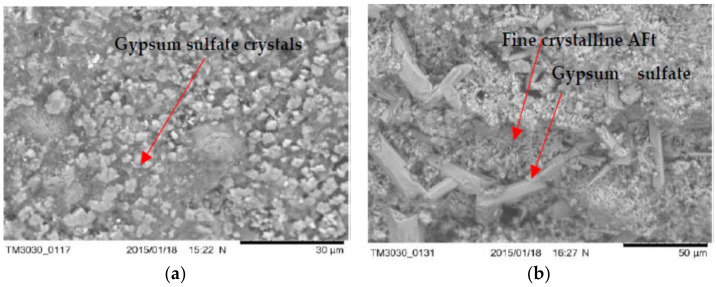
General view of the microstructure after exposure to the environment with 2% Na_2_SO_4_ (60 days) of: (**a**) LWC R1 with GEGA 2 mm, 25%, GEGA 4 mm, 25%, GAA 8 mm, 50%, and with the additive: fly ash (**b**) LWC R2 with GEGA 2 mm, 25%, GEGA 4 mm, 25%, and GAA 8 mm, 50% without fly ash.

Examples of microscopic images (SEM) of concrete samples (R1 ÷ R4) with LWA (GEGA and GAA) after 60 days of exposure to the corrosive environment are shown in Figure 12, Figure 13 and Figure 14.

The obtained results of the tests of the microstructure of the analyzed LWC (R1÷ R4) with the participation of different amounts of LWA (GEGA and GAA) suggest that in all cases, corrosion products (such as fine and coarse crystalline of ettringite—Aft and crystals of gypsum sulfate—CaSO_4_) were visible. Their amount depends on the composition of the LWC. For example, after 60 days of exposure to a 2% HCl solution, changes in the microstructure in terms of the amount of precipitated ettringite in LWC R1 with the participation of the GEGA aggregate (2 mm, 25% and 4 mm, 25%) and GAA (8 mm, 50%) were visible, both in the case of the utilization of fly ash for LWC R2 with GEGA 2 mm, 25%, GEGA 4 mm, 25%, and GAA 8 mm, 50% and without the addition of fly ash (Figure 12a,b). In the case of LWC R2, the lack of a pozzolanic mineral addition resulted in an increase in the degree of precipitation of a larger amount of ettringite crystals in the microstructure. The appearance of a corrosive product of considerable volume leads to the destruction of the structure of LWC and was confirmed by the compressive strength tests carried out by the authors (Figure 10a–c). After 60 days of exposure to 2% HCl, the average compressive strength for LWC R1 constituted 19.8 MPa, and compressive strength results for the samples stored in water for 60 days was 33.2 MPa. Compressive strength of the concrete LWC R2 without fly ash, with the same proportions and the type of lightweight aggregate after 60 days was 17.1 MPa. On the other hand, the effect of exposure to an aqueous solution of 2% CH_3_COOH and 2% Na_2_SO_4_ after 60 days resulted in the precipitation of a large amount of fine crystalline ettringite over a large area in the structure of LWC R2, as presented in Figure 13a,b in relation to LWC R4 with the participation of GEGA 2 mm, 50% and GEGA 4 mm, 50% with the participation of fly ash. In the case of the microstructure of concrete (R4), clusters of hydrated aluminum-calcium sulfate (Ca₆Al₂(SO₄)₃(OH)₁₂·26H₂O) with a well-developed crystal structure are visible (Figure 14a,b). The use of pozzolanic mineral fly ash additive in LWC increased the resistance to the effects of a weak acid solution and salt. If cement alone is used, for example, in LWC R2 and R3, the effect is lowering the pH of the pore solution in the concrete structure due to the pozzolanic reaction and the consumption of calcium hydroxide Ca(OH)_2_ of the present cement hydration product. The consequence of this phenomenon is an increase in the diffusion and the degree of penetration of corrosive solutions into the structure of LWC.

## 4. Conclusions

The appropriate durability of LWC subjected to a corrosive environment depends on the composition of the concrete: the type and amount of cement, the value of water/cement ratio, the type of aggregate, the type and number of concrete additives, and water/binder ratio. The selection and the right proportion should ensure a tight matrix in the LWC. Shaping the tightness of concrete in a particular LWC (e.g., by limiting the amount of water in the concrete mix or the use of cement with mineral additives) should primarily result in limiting the capillary porosity of the cement grout. Based on the tests carried out for LWC and presented in this work, the following conclusions can be drawn:

1. The use of a mixture of lightweight aggregate (GEGA and GAA) with cement and mineral additive such as fly ash allows a higher compressive strength of LWC over a long-term period (365 days) to be obtained compared to concrete with cement alone.
2. The level of penetration of aggressive corrosive solutions depends on the open porosity of lightweight aggregates and the type of mineral additives.
3. The presented test results for resistance to aggressive environments for LWC (R1 ÷ R4) with an aggregate (GEGA and GAA), modified with the addition of fly ash (R1, R4), indicate the influence of the LWC composition on the average compressive strength, even though the samples had been previously cured for a year (365 days) under standard conditions (temperature 20 ± 2 °C and humidity ≤95%). After a one year period, the samples were exposed to aggressive environments.
4. The use of GAA aggregate in the amount of 50% of the total aggregate volume in LWC increased the strength of concrete by approx. 50%.
5. LWC containing 100% of the total aggregate volume in the concrete is characterized by a higher resistance to corrosive environments by 8%.
6. In general, the corrosion resistance decreases as the water/binder ratio increases.
7. The introduction of silica fly ash or a mixture of fly ash as a partial replacement for cement causes significant changes in the chemical composition of the pore solution phase in the concrete structure with the use of lightweight aggregates.
8. Microstructure tests (SEM) show that the type of mineral additive affects the degree of corrosion resistance of LWC. The highest corrosion resistance, considering the change in the average compressive strength after 60 days of exposure to a corrosive environment, is demonstrated by the concrete with a mixture of lightweight aggregate (GEGA and GAA) and fly ash.
9. Concrete with lightweight aggregate (GEGA) and fly ash has a higher effect of corrosion resistance. The observed phenomenon may be the result of a slow pozzolana reaction as well as a slow process of filling the voids in the concrete structure with the products of the binding reaction.

In the initial stage of concrete maturation, it results in a decrease in compressive strength, but in later stages, compressive strength gradually increases.

10. The use of lightweight aggregates (GEGA and GAA), regardless of their mixing ratio and grain size, enables the migration of aggressive corrosive solutions, resulting in the precipitation of corrosive products such as ettringite. Its amount probably depends on the degree of sealing of the interfacial transition zone and the matrix as well as the lightweight aggregate absorption mechanism.

## Figures and Tables

**Table 1:** Chemical composition and physical properties of the binders.

Binder	Setting Start Time (min)	Setting End Time (min)	Compressive Strength (MPa)			Blaine Fineness (cm^2^/g)			Loss on Ignition (%)		Water Demand (%)	
			2 d		28 d							
CEM I 42.5R	165	205	26.5		57.4	3384			3.5		27.0	
	Content (%)											
	SiO_2_	Al_2_O_3_	Fe_2_O_3_	CaO	MgO	SO_3_	Na_2_O	K_2_O		TiO_2_		Cl^(−)^
CEM I 42.5R	21.5	6.10	3.3	63.5	1.1	2.9	0.17	0.66		0.24		0.075
Fly ash	54.8	24.4	6.6	4.2	3.0	0.5	1.10	0.36		1.20		0.055

**Table 2:** Elemental composition of the GEGA and GAA aggregates.

Aggregate Type	Content (%)								
	SiO_2_	Al_2_O_3_	Fe_2_O_3_	CaO	MgO	SO_3_	Na_2_O	K_2_O	Loss on Ignition
GEGA	63.33	0.74	–	14.19	2.98	0.32	13.35	0.57	4.53
GAA	52.82	24.28	7.5	4.5	3.19	0.43	–	0.2	7.1

**Table 3:** Physical properties of the aggregates by [11,31,33].

Property		GEGA 2 mm	GEGA 4 mm	GAA 8 mm
Water absorption WA_24_	(%)	15.2	17.8	16.5
Volume density *ρ*_a_	(kg/m^3^)	380	350	1350
Open porosity P_o_	(%)	37	42	37
Crumble indicator *X_r_*	(%)	22.3	25.9	17.8
pH after 24 h	(–)	11.9	11.9	11.1
Pore radius	(nm)	1.55–3.71	1.69–3.70	1.32–2.83
Pore volume	(cm^3^/g)	1.02–7.56 × 10^−3^	1.25–8.54 × 10^−3^	0.99–6.67 × 10^−3^

**Table 4:** Content of the components of the concrete mixture with GEGA and GAA.

Materials	Density (kg/dm^3^)	R1	R2	R3	R4
		Mass (kg/m^3^)			
CEM I 42.5 R	3.10	350	400	450	450
Fly ash	2.05	70	0	0	70
GEGA 2 mm	3.80	60	64	125	116
GEGA 4 mm	3.50	53	60	120	116
GAA 8 mm	1.35	450	450	0	0
Water	1.00	194	180	180	180
Superplasticizer	1.07	2.8	3.2	3.6	3.6
(w/c) *	(–)	0.55	0.45	0.40	0.40
(w/b) **	(–)	0.50	0.45	0.40	0.35

* w/c = water/cement; ** w/b = water/(cement + fly ash).

## Data Availability

Data is contained within the article.
